# Network pharmacology reveals the multiple mechanisms of Xiaochaihu decoction in the treatment of non-alcoholic fatty liver disease

**DOI:** 10.1186/s13040-020-00224-9

**Published:** 2020-08-27

**Authors:** Qichao Hu, Shizhang Wei, Jianxia Wen, Wenwen Zhang, Yinxiao Jiang, Caiyan Qu, Junbao Xiang, Yanling Zhao, Xi Peng, Xiao Ma

**Affiliations:** 1 School of Pharmacy, Chengdu University of Traditional Chinese Medicine, Chengdu, 611137 China; 2 Department of Pharmacy, Fifth Medical Center of PLA General Hospital, Beijing, 100039 China; 3 School of Clinical Medicine, Chengdu University of Traditional Chinese Medicine, Chengdu, 611137 China

**Keywords:** Network pharmacology, NAFLD, Xiaochaihu decoction, Immunity regulation, Metabolism regulation, Oxidative stress regulation

## Abstract

**Background:**

Non-alcoholic fatty liver (NAFLD) is a chronic disease worldwide, which poses a huge threat to human health. Xiaochaihu decoction is a well-known traditional Chinese medicine prescription. It has been proven effective in treating NAFLD but its mechanism is still unclear.

**Objective:**

Multiple mechanisms of Xiaochaihu decoction are explored by identifying and connecting potential targets and active ingredients in the treatment of NAFLD.

**Methods:**

Active ingredients and related targets of seven herbs were collected from TCMSP database. The related targets of NAFLD were obtained from Genes cards database, TDD and OMIM database. The intersected targets of disease targets and drug targets were input into STRING database to construct protein-protein interaction network. DAVID database was used for GO enrichment analysis and KEGG enrichment analysis.

**Results:**

After screening and removal of duplicates, a total of 145 active ingredients and 105 potential targets were obtained. PPI network manifested that AKT1, IL6, JUN MAPK8 and STAT3 were the key target proteins. The results of GO enrichment analysis mainly involved cytokine receptor binding, cytokine activity, and heme binding. The results of KEGG analysis suggested that the mechanism mainly involved in AGE-RAGE signaling pathway in diabetic complications, Hepatitis C, fluid shear stress and atherosclerosis. The signaling pathways were further integrated as network manner, including AGE-RAGE signaling pathway in diabetic complications, Fluid shear stress and atherosclerosis, Insulin resistance, HIF-1 signaling pathway, Th17 cell differentiation and IL-17 signaling pathway. The network contained immunity regulation, metabolism regulation and oxidative stress regulation.

**Conclusion:**

Xiaochaihu decoction plays a key role in the treatment of NAFLD with multiple targets and pathways. Immunity regulation, metabolism regulation and oxidative stress regulation consist of the crucial regulation cores in mechanism.

**Graphical abstract:**

Design and workflow of this study 
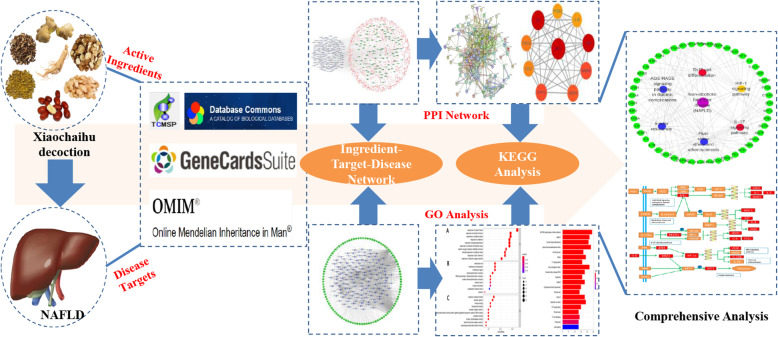

## Introduction

Non-alcoholic fatty liver disease (NAFLD) referring to the excessive fatty liver accumulation, is one of the most rapidly increasing liver diseases all over the world [1]. In decades, with the increase of obesity and other related factors, the incidence of NFALD has shown explosive growth, which makes it a cosmopolitan chronic disease with complicated manifestations [2, 3]. However, it stays unknown to reveal the mechanism of NAFLD due to the complicated process. Second-hit theory of the pathogenesis in NAFLD is currently recognized. The first hit refers to the accumulation of large amounts of fat in the liver’s parenchymal cells, which is related to insulin resistance. Increasing the concentration of insulin will cause the decomposition of peripheral adipose tissue, which causes the liver to take up more free fatty acids from the blood. At the same time, the β-oxidation of free fatty acids will be inhibited by the increase in insulin concentration, causing peroxidation and oxidation stress increased. Lipid peroxidation and oxidative stress are the second hit, which will escalate NAFLD into NASH (non-alcoholic steatohepatitis), and finally a series of changes will occur in the liver, such as liver fibrosis, hepatitis and liver cancer [4]. Another study found that T cells and B cells participate in the progression of NASH to liver fibrosis and hepatocellular carcinoma, which indicated that oxidative stress in liver cells triggered an immune response [5]. Currently, there is a lack of effective drugs for NASH, so new drugs need to be developed.

Xiaochaihu decoction comes from ShangHanLun, a well-known book in traditional Chinese medicine and is widely used in clinical practice with the effect on liver protection [6]. Many researches have demonstrated that Xiaochaihu decoction has significant effects in the treatment of NAFLD but its mechanism needs further exploration [7, 8]. A study from Qiu found that Xiaochaihu decoction could reduce the serum levels of AST (aspartate aminotransferase), ALT (alanine aminotransferase) and TC (liver tissue total cholesterol) to achieve the therapeutic effect in NAFLD rats model [9]. In attempt to systematically investigate the interaction between Chinese medicine and the complicated human body, network pharmacology has emerged on the basis of the rapid development in systems biology. This tech does provide a novel method for exploring the mechanism of traditional Chinese medicine in deep sight [10, 11]. Therefore, network pharmacology is used to reveal the mechanisms of Xiaochaihu decoction for treating NAFLD in this study. This study aims to provide a solid scientific basis for Xiaochaihu decoction to treat NAFLD. The method is through screening active ingredients in Xiaochaihu decoction and potential targets in treatment for NAFLD in order to establish the network of ingredient-target-disease and conduct GO as well as KEGG analysis.

## Materials and methods

### The collection and screen of active ingredients in Xiaochaihu decoction

The constituent herbs in Xiaochaihu decoction are comprised of Chaihu (*Radix Bupleuri*), Banxia (*Arum Ternatum Thunb*), Renshen (*Panax ginseng C. A. Mey.*), Gancao (*licorice*), Huangqin (*Scutellariae Radix*), Shengjiang (*Zingiber officinale Roscoe*) and Dazao (*Jujubae Fructus*). The active ingredients in Xiaochaihu Decoction were obtained from the Traditional Chinese Medicine Systems Pharmacology Database and Analysis Platform (http://tcmspw.com/tcmsp.php) with the keywords Banxia, Renshen, Gancao, Huangqin, Shengjiang and Dazao respectively. TCMSP, a unique Chinese herbal medicine system pharmacology platform comprising of 449 herbs, is capable of capturing the relationship between drugs, targets and diseases. The database includes chemical substances, targets and drug target networks, as well as related drug target networks. Oral Bioavailability (OB) is a significant pharmacokinetic parameter, which reflects the degree of drug absorption by the body. Drug-like (DL) refers to the similarity of the active ingredient with known drugs. The active ingredients with OB > 30% and DL > 0.18 are screened for further research [12, 13].

### Identification of potential targets of active ingredients and NFALD

Potential targets of active ingredients were retrieved from TCMSP database Related targets of NAFLD are obtained from Gene cards (https://www.genecards.org/), Therapeutic Target Database (TTD, http://db.idrblab.net/ttd/) [14] and Online Mendelian Inheritance in Man (OMIM, https://www.omim.org/).Gene cards and OMIM are online websites integrating the information about genes, disease and proteins and used for finding related targets of NAFLD. In addition, UniProt (https://www.uniprot.org) is used for finding gene names and Uniprot numbers of all targets [15].

### Construction of ingredient-target-disease network

In order to identify the corresponding relationships between the active ingredients and potential targets, we imported the active ingredients and potential targets into Cytoscape 3.7 to construct ingredient-target-disease network.

### Construction of protein-protein interaction network

STRING (http://string-db.org/cgi/input.pl) is an online database widely used for constructing protein-protein interaction (PPI) network [16] STRING database will score each interaction between target proteins. The higher score is, the higher confidence between target proteins is. Following operations are conducted, selecting “multiple proteins”, inputting the target genes of Xiaochaihu Decoction to treat NAFLD, selecting “*Homo sapiens*” for searching and setting minimum required interaction score to high confidence (0.7). Finally, the top ten target proteins were input into Cytoscape to construct the interaction network.

### Analysis of gene ontology as well as Kyoto encyclopedia of genes and genomes

To further explore the role of the mentioned screening targets above with biological signaling pathways, the potential targets are imported into the Database for Annotation, Visualization and Integrated Discovery website (DAVID, https://david.ncifcrf.gov/) for GO and KEGG analysis. The DAVID database is an online website for elucidating the potential targets in the treatment of NAFLD in biological functions and signaling pathways [17]. The following operations are conducted, selecting identifier as official gene symbol, setting list type to gene list and limiting species to *Homo sapiens*.

## Results

### Active ingredients in Xiaochaihu decoction

A total of 1476 bioactive ingredients were retrieved from TCMSP database containing 349 types in Chaihu, 116 types in Banxia, 190 types in Renshen, 280 types in Gancao, 143 types in Huangqin, 265 types in Shengjiang and 133 types in Dazao. According to OB > 30% and DL > 0.18, a total of 214 active ingredients were screened. It included 17 types in Chaihu, 13 types in Banxia, 22 types in Renshen, 92 types in Gancao, 36 types in Huangqin, 5 types in Shengjiang and 29 types in Dazao. After removal of duplicates, a total of 145 ingredients were stored for further study.

### Potential target genes of Xiaochaihu decoction and NAFLD

A total of 1181 target genes of Xiaochaihu decoction were attained from TCMSP database. Furthermore, a total of 1445 target genes of NAFLD were obtained from Genes cards, TDD and OMIM database.

### Construction of ingredient-target-disease interactive network

**Fig. 1:**
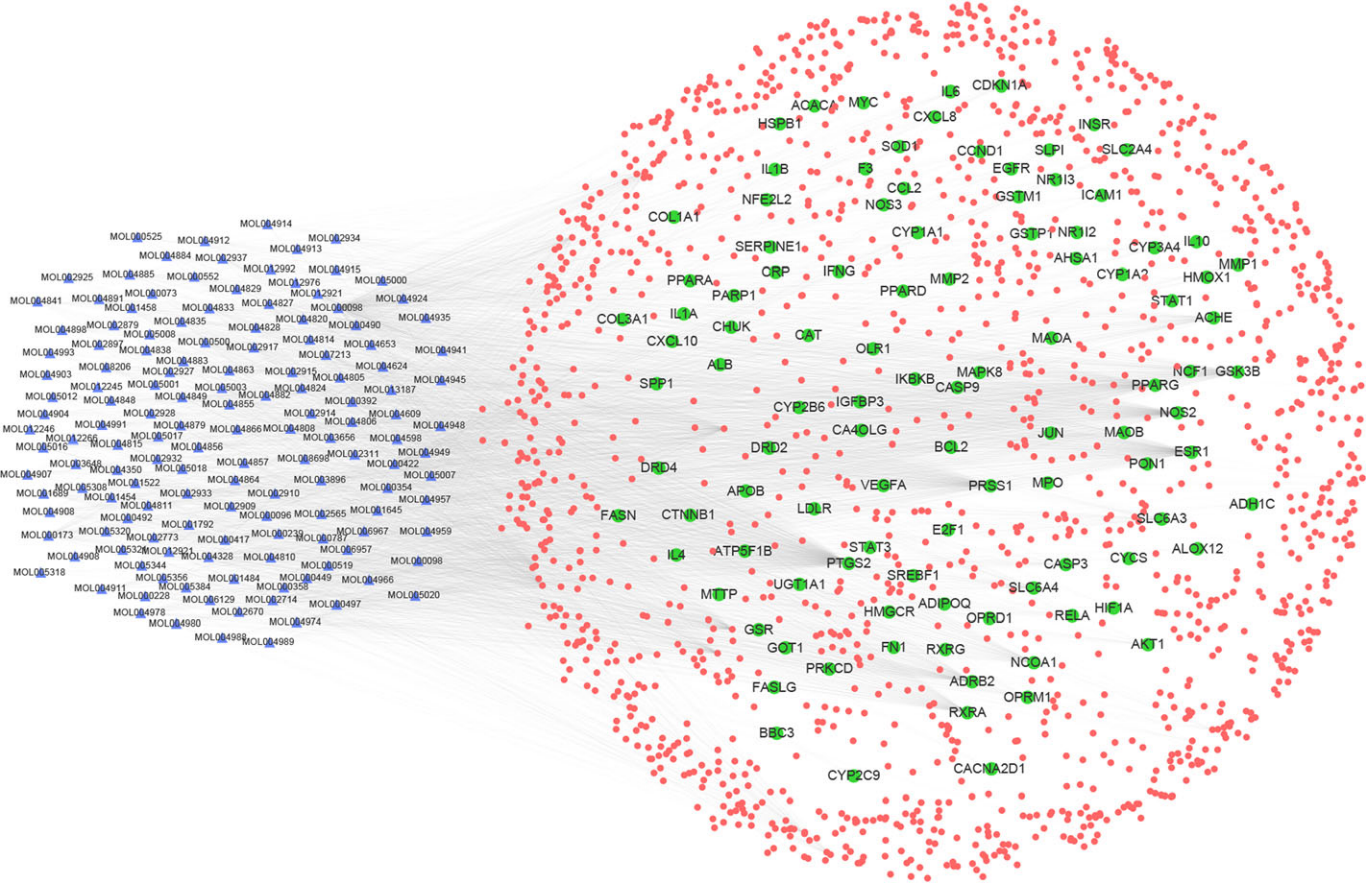
The network of ingredient-target-disease relation (The blue triangle nodes represent possible active ingredients in Xiaochaihu decoction, the red round nodes represent the targets of drugs and the disease, and the green round nodes represent the common targets of drugs and disease)

**Fig. 2:**
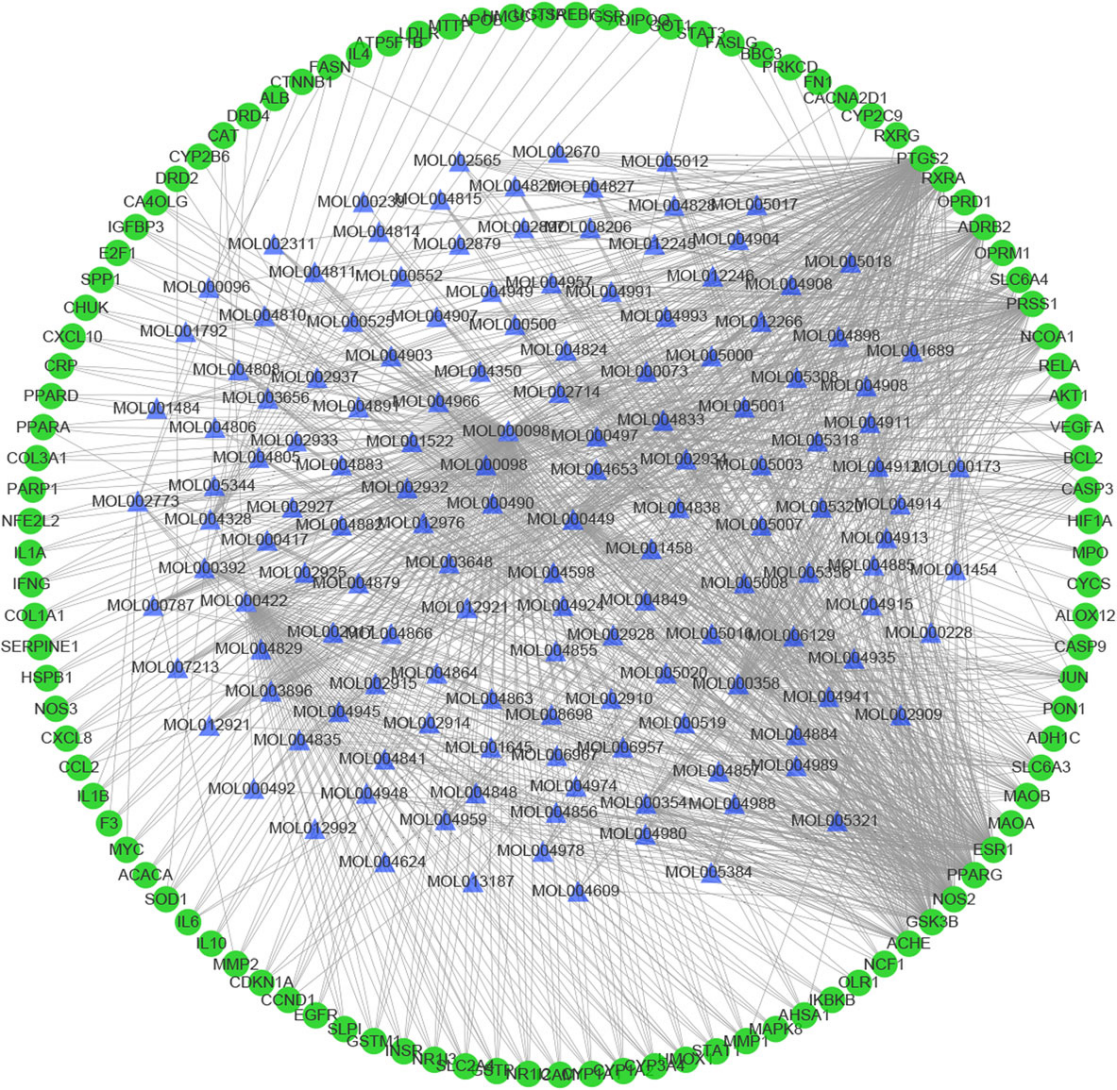
The network of ingredients and common targets of drugs and the disease (The blue triangle nodes represent possible active ingredients in Xiaochaihu decoction and the green round nodes represent the common targets of drugs and disease)

The ‘component-target-disease’ interactive network was established in order to comprehensively reveal the possible effective ingredients and potential targets of Xiaochaihu decoction against NAFLD. Moreover, network pharmacological analysis of the above data was conducted to perform visual analysis according to relevant parameters of each target. The network displayed 1515 edges and 1727 nodes with systematic 1582 targets and 145 ingredients. The nodes which possessed greater degree value and more edges played more significant roles of regulation in this network (Fig. 1). Accordingly, the network of ingredients with common targets of drugs and the disease was established to show the interactions between active ingredients and potential targets directly. There were 1750 edges and 250 nodes containing 145 ingredients and 105 common targets of drugs as well as disease in the network. The 105 common targets were regarded as potential targets in Xiaochaihu decoction for the treatment of NAFLD (Fig. 2). Degree represented the total number of routes connected to this node by other nodes in the network. The higher value, the more important corresponding ingredient or target was. After analysis with the Network Analyzer plug-in in Cytoscape software, 10 key active ingredients and 10 main targets in were listed in Tables 1 and 2 respectively. Table 1 showed that the ingredient with the highest degree was quercetin (degree = 384). The following compounds were kaempferol (degree = 168), stigmasterol (degree = 96), beta-sitosterol (degree = 90) and so on. In terms of targets, PTGS2 (degree = 161) was the most interacting ligands. The following targets were ESR1 (degree = 98), NOS2 (degree = 93), PPARG (degree = 90) and so on (Table 2). As a result, it was recognized that Xiaochaihu decoction achieved the effect on NAFLD mainly through the active ingredients acting on the key targets above.

**Table 1:** Active ingredients of Xiaochaihu decoction in the treatment of NAFLD

Molecular ID	Ingredient	Degree	Source	OB(%)	DL
MOL000098	quercetin	384	Chaihu, Dazao, Gancao	46.45	0.39
MOL000422	kaempferol	168	Chaihu, Gancao, Renshen	41.88	0.26
MOL000449	Stigmasterol	96	Banxia, Chaihu, Dazao, Huangqin, Renshen	43.83	1.44
MOL000358	beta-sitosterol	90	Banxia, Dazao, Huangqin, Renshen, Shengjiang	36.91	1.32
MOL000173	wogonin	48	Huangqin	30.68	0.79
MOL000354	isorhamnetin	48	Chaihu,Gancao	49.6	0.31
MOL002714	baicalein	48	Banxia,Huangqin	33.52	0.63
MOL004328	naringenin	42	Gancao	59.29	0.28
MOL000392	formononetin	32	Gancao	69.27	0.21
MOL002773	beta-carotene	32	Dazao	37.18	0.58

**Table 2:** The main targets of Xiaochaihu decoction in the treatment of NAFLD

UniProtKB	Target	Degree
P35354	PTGS2	161
P03372	ESR1	98
P35228	NOS2	93
P37231	PPARG	90
P07477	PRSS1	88
P07550	ADRB2	73
P49841	GSK3B	67
P19793	RXRA	62
Q15788	NCOA1	45
P22303	ACHE	38

*Notes*: *PTGS2* Prostaglandin-endoperoxide Synthase 2, *ESR1* Estrogen Receptor 1, *NOS2* Nitric Oxide Synthase 2, *PPARG* Peroxisome Proliferator Activated Receptor Gamma, *PRSS1* serine protease 1, *ADRB2* Adrenoceptor Beta 2, *GSK3B* Glycogen Synthase Kinase 3 Beta, *RXRA* Retinoid X Receptor Alpha, *NCOA1* Nuclear Receptor Coactivator 1, *ACHE* Acetylcholinesterase

### Outcomes of PPI network

**Fig. 3:**
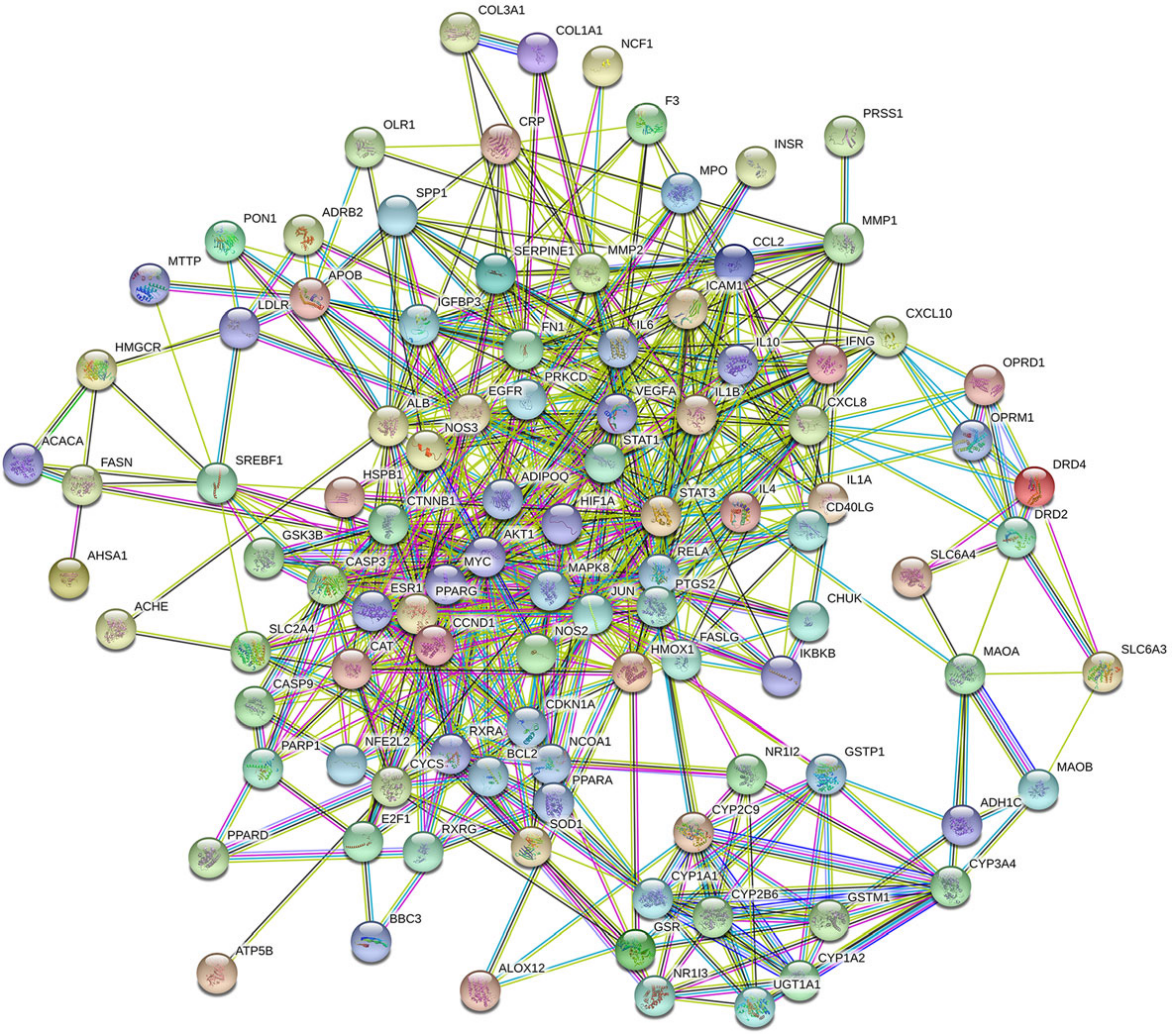
PPI network of potential targets of Xiaochaihu decoction for the treatment of NAFLD

**Fig. 4:**
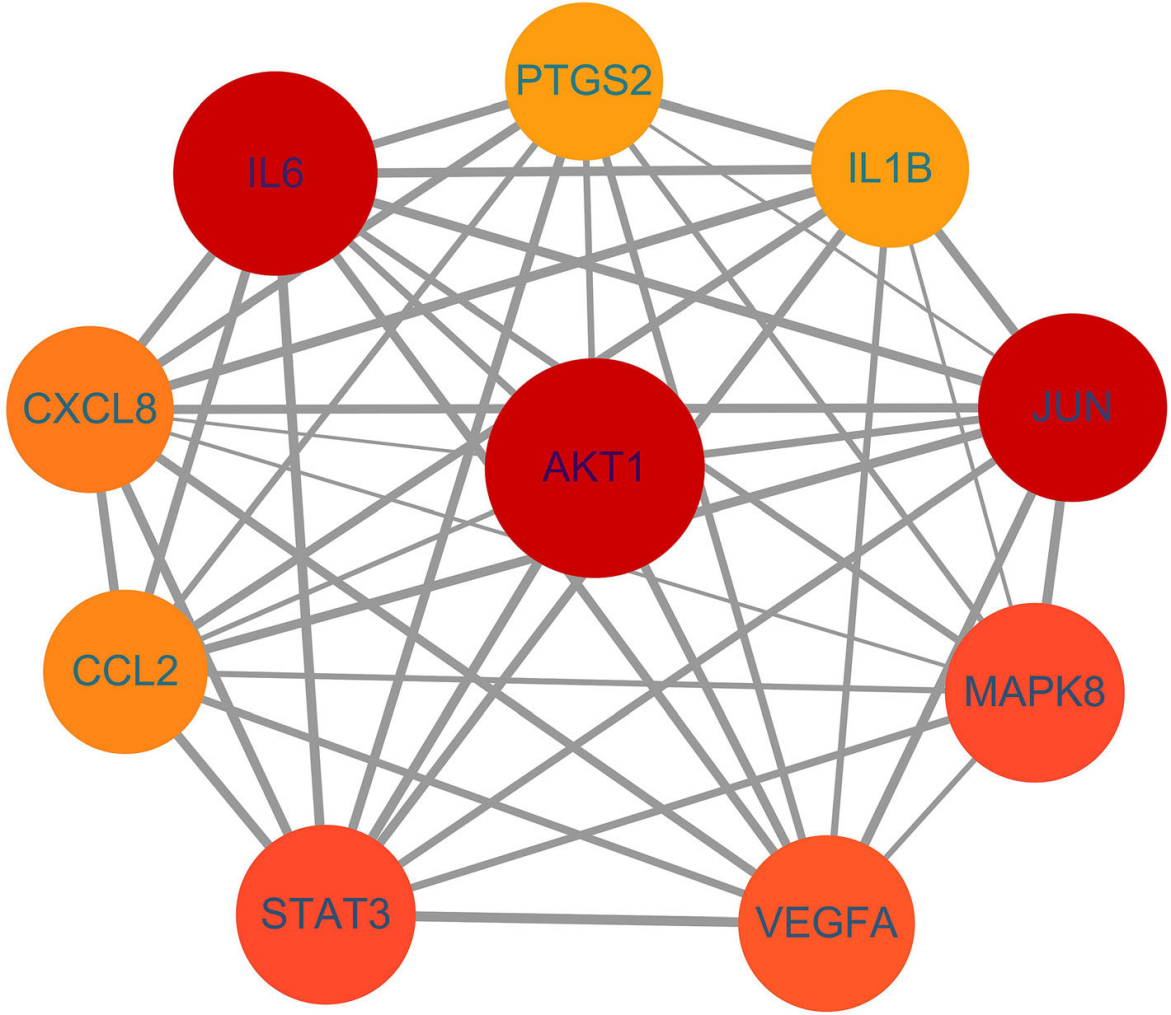
The interaction network of identified targets (AKT1: AKT Serine/Threonine Kinase 1; IL-6: Interleukin 6; JUN: Jun Proto-oncogene; MAPK8: Mitogen-activated Protein Kinase 8; STAT3: Signal Transducer and Activator of Transcription 3; PTGS2: Prostaglandin-endoperoxide Synthase 2; CXCL8: C-X-C Motif Chemokine Ligand 8; CCL2: C-C Motif Chemokine Ligand 2; IL1B: Interleukin 1 Beta; VEGFA: Vascular Endothelial Growth Factor A)

STRING was used for constructing PPI network to explore the mechanism of target genes of Xiaochaihu decoction for the treatment of NAFLD according to the high confidence (0.7) on protein level. There were 105 nodes and 608 edges in the network (Fig. 3). The average node degree was 13 and the average local clustering coefficient was 0.539. The top ten target proteins with the combined score more than o.997 were identified to be visualized by Cytoscape. The outcomes demonstrated that the target protein levels of AKT1, IL6, JUN, MAPK8 and STAT3 were excessively crucial in Xiaochaihu decoction for NAFLD (Fig. 4).

### Results of GO and KEGG enrichment analysis

#### GO enrichment analysis

**Fig. 5:**
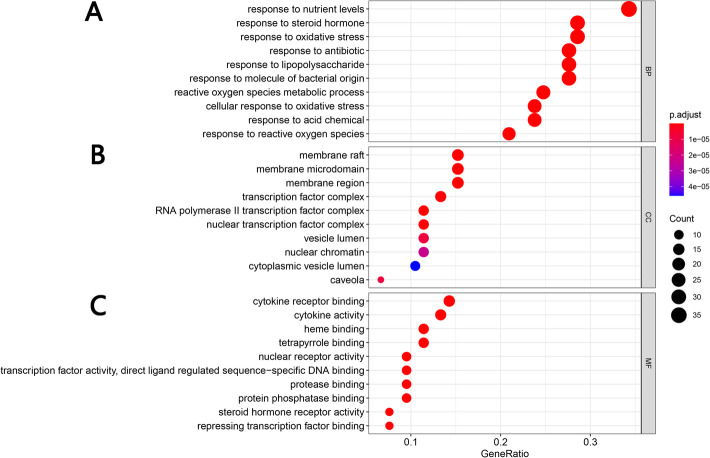
The top 10 of GO enrichment analysis (A Representative bubble plots of biological progress of core targets. B Representative bubble plots of cellular components of core targets. C Representative bubble plots of molecular function of core targets. Gene Ratio = count / set size)

The DAVID database was used for GO analysis of Xiaochaihu decoction with the potential targets for NAFLD. 651 GO entries in total were acquired based on *P* value (*P* < 0.05). It included 496 of biological progress, 100 of molecular function and 55 of cellular components. In biological progress, potential targets were mainly concentrated in response to nutrient levels, response to steroid hormone, response to oxidative stress and so on (Fig. 5a). In cellular components, potential targets were mainly concentrated in membrane raft, membrane microdomain, membrane region, transcription factor complex and so on (Fig. 5b). In terms of molecular function, potential targets were mainly enriched in cytokine receptor binding, cytokine activity, heme binding, tetrapyrrole binding and so on (Fig. 5c).

#### KEGG enrichment analysis

**Fig. 6:**
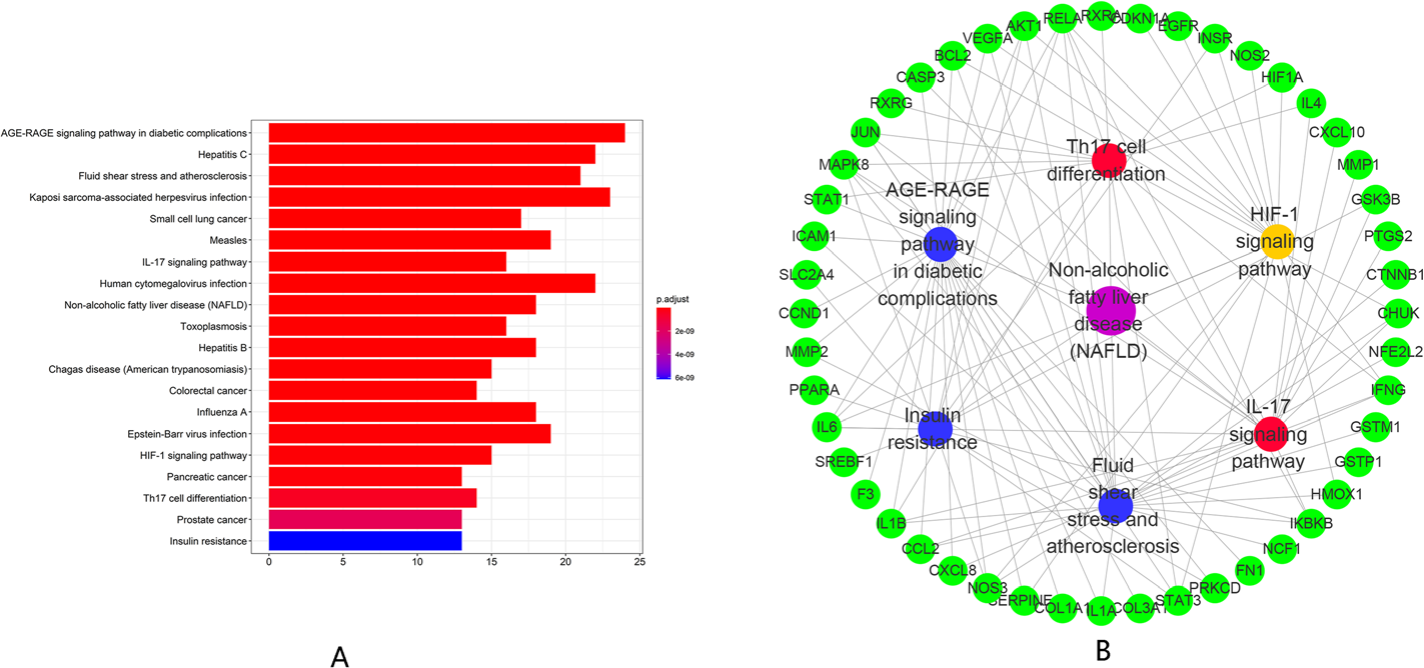
Results of KEGG Analysis. **a** The top 20 signaling pathways from KEGG analysis; **b** The disease-pathway-target interaction network of Xiaochaihu decoction for NAFLD (The purple node represents NAFLD, the yellow nodes represent pathways related to oxidative stress, the red nodes represent pathways related to immunity, the blue node represents the pathway related to metabolism and the green nodes represent targets)

DAVID was used to conduct the KEGG enrichment analysis of Xiaochaihu decoction for NAFLD (*P* < 0.05). A total of 148 pathways were obtained. The top 20 pathways in KEGG enrichment analysis was demonstrated according to *P* value (Fig. 6a). Moreover, AGE-RAGE signaling pathway in diabetic complications, hepatitis C, fluid shear stress and atherosclerosis and Kaposi sarcoma-associated herpesvirus infection were the representatives. Furthermore, they were mainly involved in immunity, cancer, metabolism and oxidative stress according to the function of these top 20 pathways (Table 3).

**Table 3:** Information of potential targets and signaling pathways

ID	Pathway	*P* value	Counts
hsa04933	AGE-RAGE signaling pathway in diabetic complications	3.12E-25	24
hsa05160	Hepatitis C	7.08E-18	22
hsa05418	Fluid shear stress and atherosclerosis	1.16E-17	21
hsa05167	Kaposi sarcoma-associated herpesvirus infection	2.70E-17	23
hsa05222	Small cell lung cancer	5.79E-16	17
hsa05162	Measles	2.88E-15	19
hsa04657	IL-17 signaling pathway	1.75E-14	16
hsa05163	Human cytomegalovirus infection	2.20E-14	22
hsa04932	Non-alcoholic fatty liver disease (NAFLD)	1.72E-13	18
hsa05145	Toxoplasmosis	3.04E-13	16
hsa05161	Hepatitis B	7.49E-13	18
hsa05142	Chagas disease (American trypanosomiasis)	1.15E-12	15
hsa05210	Colorectal cancer	1.63E-12	14
hsa05164	Influenza A	1.73E-12	18
hsa05169	Epstein-Barr virus infection	3.02E-12	19
hsa04066	HIF-1 signaling pathway	3.13E-12	15
hsa05212	Pancreatic cancer	5.57E-12	13
hsa04659	Th17 cell differentiation	3.54E-11	14
hsa05215	Prostate cancer	1.38E-10	13
hsa04931	Insulin resistance	5.44E-10	13

## Discussion

As one of the most common liver diseases, the incidence of NAFLD is increasingly rising worldwide and its prevalence is approximately 25.24% in liver disease. Patients with NAFLD are more likely to suffer from cardiovascular and tumor diseases and have a lower survival rate compared with ordinary patients [18]. Xiaochaihu decoction consists of 7 herbs, including Chaihu (*Radix Bupleuri*), Banxia (*Arum Ternatum Thunb*), Renshen (*Panax ginseng* C. A. Mey.), Gancao (*licorice*), Huangqin (*Scutellariae Radix*), Shengjiang (*Zingiber officinale Roscoe*) and Dazao (*Jujubae Fructus*). To date, many studies have discovered that Chaihu and its prescription bring out positive therapeutic effects on NAFLD [19, 20]. Ginsenoside Rg1 from Renshen is confirmed to treat NAFLD via suppressing inflammasome activation [21]. In addition, baicalin and Paeoniflorin from Huangqin are also appeared to ameliorate NAFLD [22, 23]. In order to explore the multiple mechanisms of Xiaochaihu decoction, TCMSP database was used to obtain the effective active ingredients and potential targets in this study. Moreover, the NAFLD related disease targets were searched in the Gene cards database, TTD and the OMIM database. Common targets were obtained through gene mapping. The ‘ingredient-target-disease’ interaction network was constructed by Cytoscape software.

### Analysis of active ingredients

In the network of ingredient-target-disease, key ingredients with higher degree includes quercetin, kaempferol, stigmasterol and beta-sitosterol. Quercetin is a widely existing flavonoid in nature. A recent research showed that quercetin had anti-oxidant, anti-inflammatory and immunomodulatory effects in NAFLD treatment [24]. A clinical trial found that treatment with quercetin could change the hematological parameters of patients with NAFLD with increasing of RBC and decreasing of ferritin [25]. Kaempferol has a variety of pharmacological effects such as anti-cancer, anti-oxidation, anti-viral, anti-inflammatory, antibacterial activities. It can also enhance the immunity and attenuate liver fibrosis via inhibiting activin receptor-like kinase 5 [26, 27]. Both beta-sitosterol and stigmasterol are components of phytosterols, have a wide range of pharmacological effects such as anti-inflammatory, antibacterial, analgesic, and anti-cancer. Moreover, beta-sitosterol and stigmasterol were proved to be effective in NAFLD mainly by regulating lipid metabolism [28].

### Analysis of potential targets

The target with the highest degree was PTGS2. The following targets were ESR1, NOS2, PPARG in the network of ingredient-target-disease. PTGS2 is an oxidative stress-related factor related to prostaglandin biosynthesis related to inflammation and mitosis. He found that down-regulation of PTGS2 mRNA was related to the repair of drug-induced liver injury in mice [29]. ESR1 is an estrogen receptor expressed by hepatocytes. NOS2 is an isozyme of NOS which plays an important role in inflammation, tumor and cell damage. PPARG could not only inhibit the expression of inflammatory factors such as TNF-α and IL-1, but also regulate adipocyte differentiation as well as lipid metabolism. This activity might contribute to the prevention against NAFLD [30]. Above all, the key ingredients containing quercetin, kaempferol, stigmasterol and beta-sitosterol might be closely related to the regulation of PTGS2, ESR1, NOS2 and PPARG during Xiaochaihu decoction treating NAFLD.

### Analysis of PPI network

The target protein levels of AKT1, IL6, JUN, MAPK8 and STAT3 were excessively expressed in PPI network. This result indicated the series of targets of Xiaochaihu decoction treating NAFLD. An animal model research has proven that the decreased expression of AKT1 resulted in FoxO1 inhibition and further achieved the effect of ameliorating NAFLD in diabetic rat [31]. Another research also suggested that the inhibition of AKT1-mediated ROS production suppressed the process of NAFLD to liver fibrosis conversion [32]. IL-6 is an important pro-inflammatory factor and its production has been shown to be positively correlated with the occurrence and development of NAFLD [33]. JUN including JNK, JUN B and JUN C belongs to the AP-1 family of transcription factors. The research from Yan indicated that JNK participated in mediating the process of liver inflammation and fat accumulation during NAFLD [34]. Moreover, MAPK8 was confirmed to be related to liver regeneration in mice [35]. It might contribute to the prevention against NAFLD. Yu’s study has found that up-regulation of the NF-kB and STAT3 signaling pathway could promote insulin resistance in liver cells which was potent for NAFLD prevention [36]. As a result, the comprehensive regulation of AKT1, IL6, JUN, MAPK8 and STAT3 was potential for the therapeutic effect on NAFLD with Xiaochaihu decoction.

### Analysis of signaling pathways

**Fig. 7:**
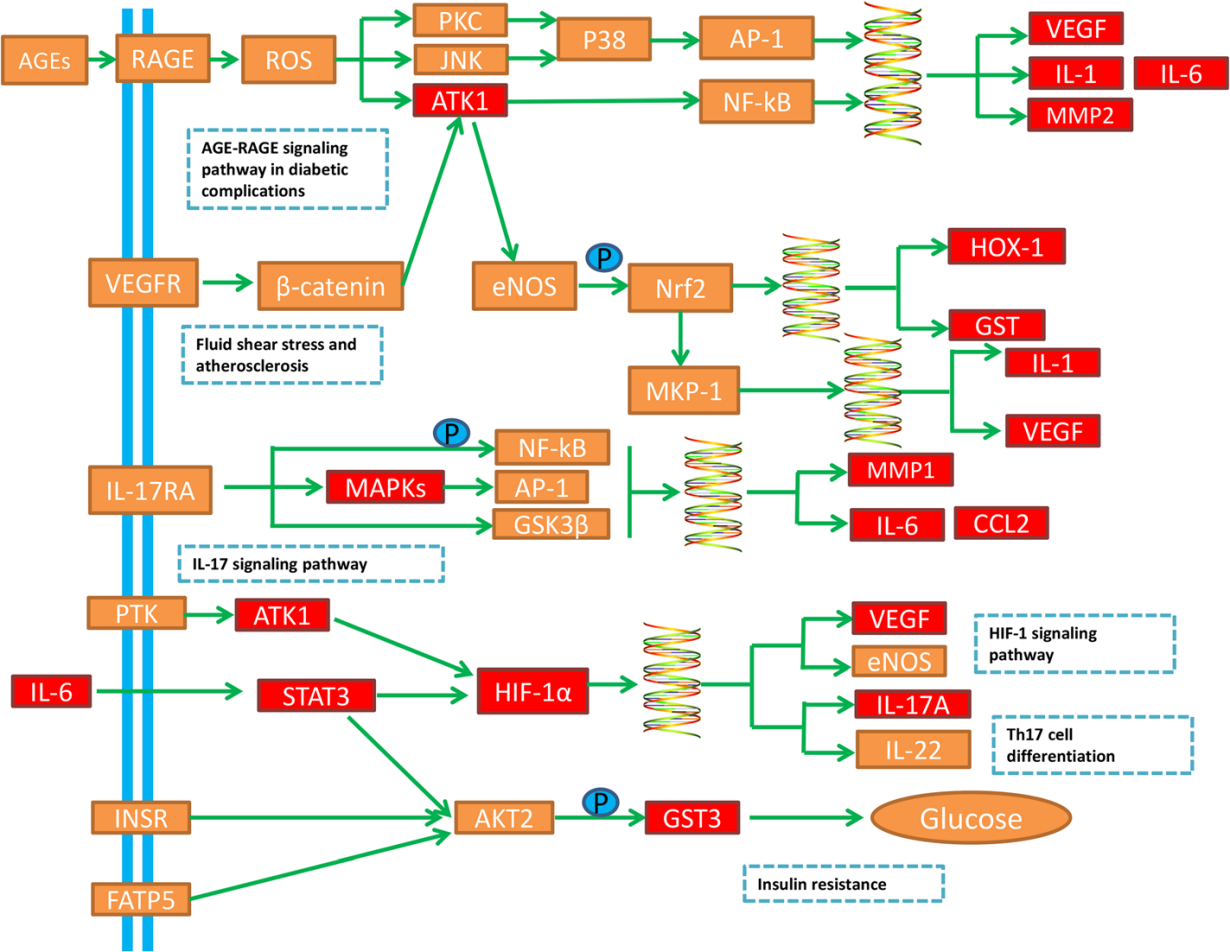
Distribution of the target proteins of Xiaochaihu decoction on the predicted pathways (The red nodes are potential target proteins of Xiaochaihu decoction, while the orange nodes are relevant targets in the pathway)

DAVID database was used for conducting KEGG analysis and GO analysis. The result of GO indicated that potential targets were mainly enriched in response to nutrient levels in biological progress, cytokine receptor binding in molecular function and membrane raft in cellular components. In addition, the results of KEGG were directly related to the NAFLD pathway. We retrieved six pathways related to liver disease as core pathways from the top 20 pathways of KEGG enrichment analysis and divided them into three aspects: metabolism, oxidative stress and immunity. As shown in Fig. 6b, the ‘disease-pathway-target’ interaction network demonstrated that active ingredients in Xiaochaihu decoction played a multi-targets and multi-pathways role in regulation of NAFLD. Furthermore, Fig. 7 demonstrates the distribution of the target proteins of Xiaochaihu decoction on the predicted pathways. The pathways related to metabolism included AGE-RAGE signaling pathway in diabetic complications, Fluid shear stress and atherosclerosis and Insulin resistance. According to previous statistics, NAFLD is a common complication of diabetes [37]. The accumulation of fat caused by NAFLD will increase insulin resistance, which will affect the liver’s export of hepatic glycogen. This process will eventually lead to disorder of lipid metabolism and aggravate the condition. Asadipooya Kamyar found that AGE-RAGE interaction could promote the fat accumulation in the liver. It will cause many complications in NAFLD including inflammation, fibrosis and insulin resistance [38]. The pathological basis of atherosclerosis is the disorder of lipid metabolism. It often occurs together with NAFLD and makes the condition worsen. A study revealed that the occurrence of NAFLD was closely related to atherosclerosis [39]. The pathways related to oxidative stress is HIF-1 signaling pathway. The second-hit theory, a currently recognized theory revealing the pathogenesis of NAFLD, stated that oxidative stress could accelerate the conversion of NAFLD into NASH. A new research also found that patients with NAFLD had significantly higher expression of HIF-1 in peripheral blood and Th17 cell differentiation compared with healthy people. Moreover, the joint examination on HIF-1 and Th17 cell might be helpful for the early diagnosis of NAFLD [40]. The pathways related to immunity include Th17 cell differentiation and IL-17 signaling pathway. A study from Su demonstrated that the levels of Th22 cell and Th17 cell were positively correlated with the degree of liver lesions in NAFLD model mice and involved in the occurrence as well as development of NAFLD [40, 41]. Generally speaking, patients with infection-induced immunodeficiency are more susceptible to NAFLD. Overall, the above pathways and their interactions may be closed relevant to NAFLD.

In this study, network pharmacology systematically uncovered the key targets, active ingredients and the crucial signaling pathways of Xiaochaihu decoction for the treatment of NAFLD. It also provided a direction and evidence for future researches. Nonetheless, there still exists the limitation in this study. The source, performance and dosage of traditional Chinese medicine in actual application are not considered in this study. This is whatever so important during research. Moreover, there are many other variable factors should be noticed including NAFLD models in different species. In addition, network pharmacology is based on the data in the existing literature. In other words, if the data in the existing literature is biased, the results of network pharmacology will be inaccurate, which is the limitation of prediction. The treatment of NAFLD by natural products is often achieved through multiple targets and multiple pathways. Therefore, more researches approaches are ought to carry out in the future to further reveal the mechanisms of well-known formula and the compounds derived from these for various liver diseases [42].

## Conclusion

In summary, Xiaochaihu decoction plays a key role in the treatment of NAFLD with multiple targets and pathways. Immunity regulation, metabolism regulation and oxidative stress regulation consist of the crucial regulation cores in this mechanism.

## Data Availability

All data are available in the manuscript and they are showed in figures and tables.

## Abbreviations

NAFLD: Non-alcoholic Fatty Liver Disease
NASH: Non-alcoholic steatohepatitis
AST: Aspartate aminotransferase
ALT: Alanine aminotransferase
TC: Liver tissue total cholesterol
TCMSP: Traditional Chinese Medicine Systems Pharmacology
DL: Drug-likeness
OB: Oral bioavailability
DAVID: Annotation, Visualization and Integrated Discovery
GO: Gene ontology
BP: Biological process
KEGG: Kyoto Encyclopedia of Genes and Genomes
PTGS2: Prostaglandin-endoperoxide Synthase 2
ESR1: Estrogen Receptor 1
NOS2: Nitric Oxide Synthase 2
PPARG: Peroxisome Proliferator Activated Receptor Gamma
PRSS1: Serine protease 1
ADRB2: Adrenoceptor Beta 2
GSK3B: Glycogen Synthase Kinase 3 Beta
RXRA: Retinoid X Receptor Alpha
NCOA1: Nuclear Receptor Coactivator 1
ACHE: Acetylcholinesterase

## References

[1] Alwahsh SM, Gebhardt R (2017). Arch Toxicol.

[2] Kanwal F, Tapper EB, Ho C, Asrani SK, Ovchinsky N, Poterucha J (2019). Development of quality measures in cirrhosis by the practice metrics Committee of the American Association for the study of liver diseases. Hepatology..

[3] Fan JG, Kim SU, Wong VWS. New trends on obesity and NAFLD in Asia. J Hepatol. 2017. 10.1016/j.jhep.2017.06.003 European Association for the Study of the liver. 10.1016/j.jhep.2017.06.003 28642059

[4] Sutti S, Albano E (2020). Adaptive immunity: an emerging player in the progression of NAFLD. Nat Rev Gastroenterol Hepatol.

[5] Turchinovich A, Baranova A, Drapkina O, Tonevitsky A (2018). Cell-free circulating nucleic acids as early biomarkers for NAFLD and NAFLD-associated disorders. Front Physiol.

[6] Zhou Y, Jiang Y, Zheng P (2019). 1. Research Progress in traditional Chinese medicine of nonalcoholic fatty liver. J Liaoning Univ Tradit Chin Med.

[7] Shi T, Wu L, Ma W, Ju L, Bai M, Chen X (2020). Nonalcoholic fatty liver disease: pathogenesis and treatment in traditional Chinese medicine and Western medicine. Evid Based Complement Alternat Med.

[8] Ruoxuan Z, Wenliang L (2019). Research Progress in treatment of nonalcoholic fatty liver disease by Chinese medicine. J Hubei Univ Chinese Med.

[9] Qiu G, Ye F, Liu Y, Cai Y, Wang R, Sun Y, He Q (2013). The experimental study on the effects of Xiaochaihu Soup on nonalcoholic rat fatty liver. J Xi’an Jiaotong Univ Medical Sci.

[10] Liu JF, Hu AN, Zan JF, Wang P, You QY, Tan AH (2019). Network pharmacology deciphering mechanisms of volatiles of Wendan granule for the treatment of Alzheimer’s disease. Evid Based Complement Alternat Med.

[11] Niu B, Zhang H, Li C, Yan F, Song Y, Hai G (2019). Network pharmacology study on the active components of pterocypsela elata and the mechanism of their effect against cerebral ischemia. Drug Des Devel Ther.

[12] Ru J, Li P, Wang J, Zhou W, Li B, Huang C (2014). TCMSP: a database of systems pharmacology for drug discovery from herbal medicines. Aust J Chem.

[13] Ahmed SSSJ, Ramakrishnan V (2012). Systems biological approach of molecular descriptors connectivity: optimal descriptors for oral bioavailability prediction. PLoS One.

[14] Wang Y, Zhang S, Li F, Zhou Y, Zhang Y, Wang Z (2020). Therapeutic target database 2020: enriched resource for facilitating research and early development of targeted therapeutics. Nucleic Acids Res.

[15] Bateman A (2019). UniProt: a worldwide hub of protein knowledge. Nucleic Acids Res.

[16] Szklarczyk D, Morris JH, Cook H, Kuhn M, Wyder S, Simonovic M (2017). The STRING database in 2017: quality-controlled protein-protein association networks, made broadly accessible. Nucleic Acids Res.

[17] Sherman BT, Huang DW, Tan Q, Guo Y, Bour S, Liu D (2007). DAVID knowledgebase: a gene-centered database integrating heterogeneous gene annotation resources to facilitate high-throughput gene functional analysis. BMC Bioinformatics.

[18] Younossi ZM, Koenig AB, Abdelatif D, Fazel Y, Henry L, Wymer M (2016). Global epidemiology of nonalcoholic fatty liver disease—meta-analytic assessment of prevalence, incidence, and outcomes. Hepatology..

[19] Nascimbeni F, Pais R, Bellentani S, Day CP, Ratziu V, Loria P (2013). From NAFLD in clinical practice to answers from guidelines. J Hepatol.

[20] Ballestri S, Capitelli M, Fontana MC, Arioli D, Romagnoli E, Graziosi C, et al. Direct Oral Anticoagulants in Patients with Liver Disease in the Era of Non-Alcoholic Fatty Liver Disease Global Epidemic: A Narrative Review. Adv Ther; 2020: 1910-1932: https://doi.org/10.1007/s12325-020-01307-z. Springer Healthcare. 10.1007/s12325-020-01307-z PMC7467481 32285340

[21] Xu Y, Yang C, Zhang S, Li J, Xiao Q, Huang W (2018). Ginsenoside Rg1 protects against non-alcoholic fatty liver disease by ameliorating lipid peroxidation, endoplasmic reticulum stress, and Inflammasome activation. Biol Pharm Bull.

[22] Zhang J, Zhang H, Deng X, Zhang N, Liu B, Xin S (2018). Baicalin attenuates non-alcoholic steatohepatitis by suppressing key regulators of lipid metabolism, inflammation and fibrosis in mice. Life Sci.

[23] Ma X, Zhang W, Jiang Y, Wen J, Wei S, Zhao Y (2020). Paeoniflorin, a natural product with multiple targets in liver diseases—a mini review. Front Pharmacol.

[24] Zhu X, Xiong T, Liu P, Guo X, Xiao L, Zhou F (2018). Quercetin ameliorates HFD-induced NAFLD by promoting hepatic VLDL assembly and lipophagy via the IRE1a/XBP1s pathway. Food Chem Toxicol.

[25] Pasdar Y, Oubari F, Zarif MN, Abbasi M, Pourmahmoudi A, Hosseinikia M (2020). Effects of Quercetin supplementation on hematological parameters in non-alcoholic fatty liver disease: a randomized, double-blind, Placebo-Controlled Pilot Study. Clin Nutr Res.

[26] Xu T, Huang S, Huang Q, Ming Z, Wang M, Li R (2019). Kaempferol attenuates liver fibrosis by inhibiting activin receptor–like kinase 5. J Cell Mol Med.

[27] Chen J, Xuan YH, Luo MX, Ni XG, Ling LQ, Hu SJ (2020). Kaempferol alleviates acute alcoholic liver injury in mice by regulating intestinal tight junction proteins and butyrate receptors and transporters. Toxicology.

[28] Feng S, Dai Z, Liu AB, Huang J, Narsipur N, Guo G (2018). Intake of stigmasterol and β-sitosterol alters lipid metabolism and alleviates NAFLD in mice fed a high-fat western-style diet. Biochim Biophys Acta Mol Cell Biol Lipids.

[29] He Y, Liu C, He C, Zhao J, Sun Y, Xu H (2018). Protective effect of Fuzheng Yanggan mixture on drug-induced liver injury. China J Chinese Mater Medica.

[30] Yang Z, Wen J, Li Q, Tao X, Ye Z, He M (2012). PPARG gene Pro12Ala variant contributes to the development of non-alcoholic fatty liver in middle-aged and older Chinese population. Mol Cell Endocrinol.

[31] Xie X, Yan D, Li H, Zhu Q, Li J, Fang YP (2018). Enhancement of Adiponectin ameliorates nonalcoholic fatty liver disease via inhibition of FoxO1 in type 1 diabetic rats. J Diabetes Res.

[32] Wu H, Chen G, Wang J, Deng M, Yuan F, Gong J (2020). TIM-4 interference in Kupffer cells against CCL4-induced liver fibrosis by mediating Akt1/Mitophagy signalling pathway. Cell Prolif.

[33] Hendy OM, Elsabaawy MM, Aref MM, Khalaf FM, Oda AMA, El Shazly HM (2017). Evaluation of circulating zonulin as a potential marker in the pathogenesis of nonalcoholic fatty liver disease. Apmis..

[34] Yan FJ, Wang X, Wang SE, Hong HT, Lu J, Ye Q (2020). C-Jun/C7ORF41/NF-κB axis mediates hepatic inflammation and lipid accumulation in NAFLD. Biochem J.

[35] Langiewicz M, Graf R, Humar B, Clavien PA (2018). JNK1 induces hedgehog signaling from stellate cells to accelerate liver regeneration in mice. J Hepatol.

[36] Heo YJ, Choi SE, Jeon JY, Han SJ, Kim DJ, Kang Y (2019). Visfatin induces inflammation and insulin resistance via the NF-κB and STAT3 signaling pathways in hepatocytes. J Diabetes Res.

[37] Tian F, Zheng Z, Zhang D, He S, Shen J (2018). Efficacy of liraglutide in treating type 2 diabetes mellitus complicated with non-alcoholic fatty liver disease. Biosci Rep.

[38] Asadipooya K, Lankarani KB, Raj R, Kalantarhormozi M (2019). RAGE is a potential cause of onset and progression of nonalcoholic fatty liver disease. Int J Endocrinol.

[39] Stols-Gonçalves D, Hovingh GK, Nieuwdorp M, Holleboom AG (2019). NAFLD and atherosclerosis: two sides of the same Dysmetabolic coin?. Trends Endocrinol Metab.

[40] Wang R, Li D, Qi W, Sun J (2019). 1. Expression and clinical significance of HIF-1α and Th17 / Treg in peripheral blood of patients with non-alcoholic fatty liver disease. Clin J Med Off.

[41] Su S-B, Chen W, Huang F-F, Zhang J-F (2018). Elevated Th22 cells correlated with Th17 cells in patients with high liver stiffness in nonalcoholic fatty liver disease. Eur J Inflamm.

[42] Ma X, Jiang Y, Zhang W, Wang J, Wang R, Wang L (2020). Natural products for the prevention and treatment of cholestasis: a review. Phyther Res.

